# Supplementary material for: Bile Acid Metabolism Analysis Provides Insights into Vascular Endothelial Injury in Salt-Sensitive Hypertensive Rats
Source: Metabolites. 2024 Aug 16;14(8):452. doi: 10.3390/metabo14080452 (PMC11356606; doi:10.3390/metabo14080452)
Supplement: Supplementary file 1 [file metabolites-14-00452-s001.zip › Supplementary Files.pdf]

The Specific Components of Rat Feed
(Not Including the Content of NaCl)

Components	Proportion
Casein	20
Methionine	0.3
Sucrose	50
Corn Starch	15
Cellulose	5
Corn Oil	5
Multi Mineral S10001	3.5
Multidimensional V10001	1
Choline Bitartrate	0.2
Total	100

Table S1: The specific components of rat feed. NC and LSD groups fed feed with a salt content of 0.3%. HSD group fed feed with a salt content of 8%.

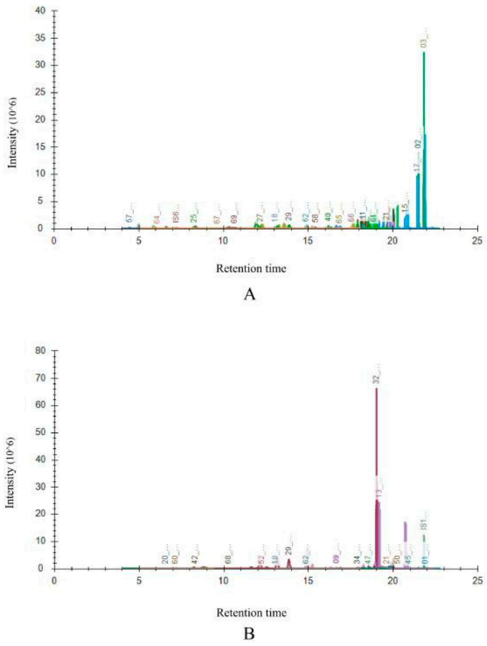

Figure S1: The extracted ion chromatographs from a standard solution and a sample of the targeted analytes. (A) standard solution (B) sample solution.

The Basal Level of Blood Pressure of Three Groups (mean ± SEM)

Group	n	SBP (mmHg)	DBP (mmHg)
**NC**	6	119±17	84±7
**LSD**	6	137±6	92±5
**HSD**	6	136±3	91±10

Table S2: The basal level (before modeling) of blood pressure of the NC, LSD and HSD groups.

The Blood Pressure of Three Groups (mean ± SEM)

Group	n	SBP (mmHg)	DBP (mmHg)
**NC**	6	119±17	86±8
**LSD**	6	143±9	101±5
**HSD**	6	206±9	150±9

Table S3: The blood pressure of the NC, LSD and HSD groups.

The Levels of Vascular Regulatory and Inflammatory Factors (mean ± SEM)

	NC	LSD	HSD
**ET-1 (pg/mL)**	1.43±0.21	1.27±0.13	1.65±0.21
**NO (μmol/L)**	27.06±7.12	35.41±13	30.03±10.1
**Ang-II (pg/mL)**	33.33±10.56	15.99±4.22	19.47±11.84
**TNF-α (pg/mL)**	25.35±12.55	16.02±1.89	26.04±3.88
**IL-6 (pg/mL)**	10.52±0.77	10.06±0.31	10.08±0.6
**IL-10 (pg/mL)**	10.32±2.28	14.18±6.29	14.14±4.2

Table S4: The levels of vascular regulatory and inflammatory factors.

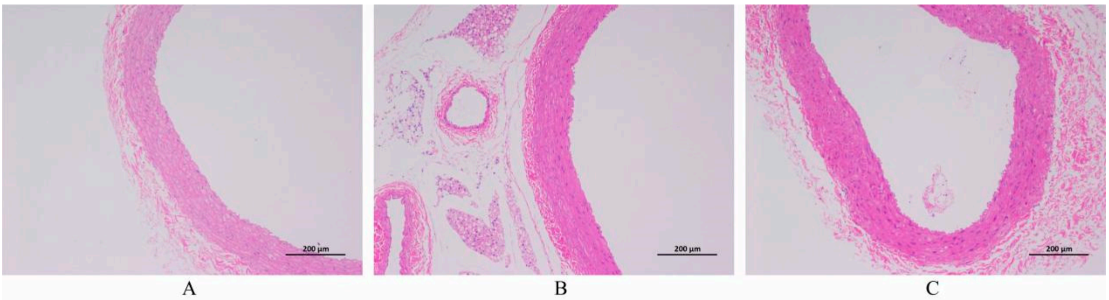

Figure S2: The histopathological observation of arterial blood vessels (H&E staining). (A) NC group. (B) LSD group. (C) HSD group. Scale bar: 200 µm.

The Detailed Information on Detected Bile Acids

Name	Abbreviation	CAS Number	Chemical formula
Isolithocholic acid	isoLCA	1534-35-6	C24H40O3
Lithocholic acid	LCA	434-13-9	C24H40O3
6-Ketolithocholic acid	6-ketoLCA	2393-61-5	C24H38O4
7-Ketolithocholic acid	7-ketoLCA	4651-67-6	C24H38O4
12-Ketolithocholic acid	12-ketoLCA	5130-29-0	C24H38O4
Apocholic acid	apoCA	641-81-6	C24H38O4
Isoursodeoxycholic acid	isoUDCA	78919-26-3	C24H40O4
Murideoxycholic acid	MDCA	668-49-5	C24H40O4
Isohyodeoxycholic acid	isoHDCA	570-84-3	C24H40O4
Ursodeoxycholic acid	UDCA	128-13-2	C24H40O4
Hyodeoxycholic acid	HDCA	83-49-8	C24H40O4
3-Epideoxycholic acid	βDCA	570-63-8	C24H40O4
Chenodeoxycholic acid	CDCA	474-25-9	C24H40O4
Deoxycholic acid	DCA	83-44-3	C24H40O4
7,12-Diketolithocholic acid	7,12-diketoLCA	517-33-9	C24H36O5
7-Ketodeoxycholic acid	7-KHCA	911-40-0	C24H38O5
12-Dehydrocholic acid	12-DHCA	2458/8/4	C24H38O5
3-Dehydrocholic acid	3-DHCA	2304-89-4	C24H38O5

Name	Abbreviation	CAS Number	Chemical formula
Ursocholic acid	UCA	2955-27-3	C24H40O5
ω-Muricholic Acid	ωMCA	6830/3/1	C24H40O5
α-Muricholic acid	α-MCA	2393-58-0	C24H40O5
β-Muricholic acid	β-MCA	2393-59-1	C24H40O5
Hyocholic acid	HCA	547-75-1	C24H40O5
Allocholic acid	ACA	2464-18-8	C24H40O5
Cholic acid	CA	81-25-4	C24H40O5
Glycolithocholic acid	GLCA	474-74-8	C26H43NO4
Glycohyodeoxycholic acid	GHDCA	13042-33-6	C26H43NO5
Glycochenodeoxycholic acid	GCDCA	640-79-9	C26H43NO5
Glycodeoxycholic acid	GDCA	360-65-6	C26H43NO5
Glycocholic acid	GCA	475-31-0	C26H43NO6
Taurolithocholic acid	TLCA	6042-32-6	C26H45NO5S
Tauroursodeoxycholic acid	TUDCA	14605-22-2	C26H45NO6S
Taurohyodeoxycholic acid	THDCA	2958/4/5	C26H45NO6S
Taurochenodeoxycholic acid	TCDCA	516-35-8	C26H45NO6S
Tauro ω-muricholic acid	TωMCA	130325-58-5	C26H45NO7S
Tauro α-Muricholic acid	T-α-MCA	25613-05-2	C26H45NO7S
Tauro β-Muricholic acid	T-β-MCA	25696-60-0	C26H45NO7S
Taurocholic acid	TCA	81-24-3	C26H45NO7S

Table S5: The Detailed information of detected bile acids.

The Bile Acid Levels of LSD and HSD Groups (mean ± SEM)

	LSD	HSD
**Glycocholic acid**	311.13±142	869.28±284.86
**Taurolithocholic acid**	10.79±3.61	32.75±12.15
**Tauroursodeoxycholic acid**	30.46±10.46	64.11±17.12
**Glycolithocholic acid**	9.46±1.8	34.45±16.55

Table S6: The significant differential bile acid levels of LSD and HSD groups.

The p-value of Correlation Analysis

	ET-1	NO	ANG-II	TNF-α	IL-6	IL-10
**Glycocholic acid**	0.011	0.144	0.473	0.006	0.550	0.953
**Taurolithocholic acid**	0.005	0.364	0.963	0.006	0.336	0.836
**Tauroursodeoxycholic acid**	0.001	0.515	0.780	0.004	0.533	0.752
**Glycolithocholic acid**	0.03	0.26	0.42	0.01	0.48	0.82

Table S7: The p-value of correlation analysis.
