# Supplementary figures and images for: Bile Acid Metabolism Analysis Provides Insights into Vascular Endothelial Injury in Salt-Sensitive Hypertensive Rats
Source: Metabolites. 2024 Aug 16;14(8):452. doi: 10.3390/metabo14080452 (PMC11356606; doi:10.3390/metabo14080452)

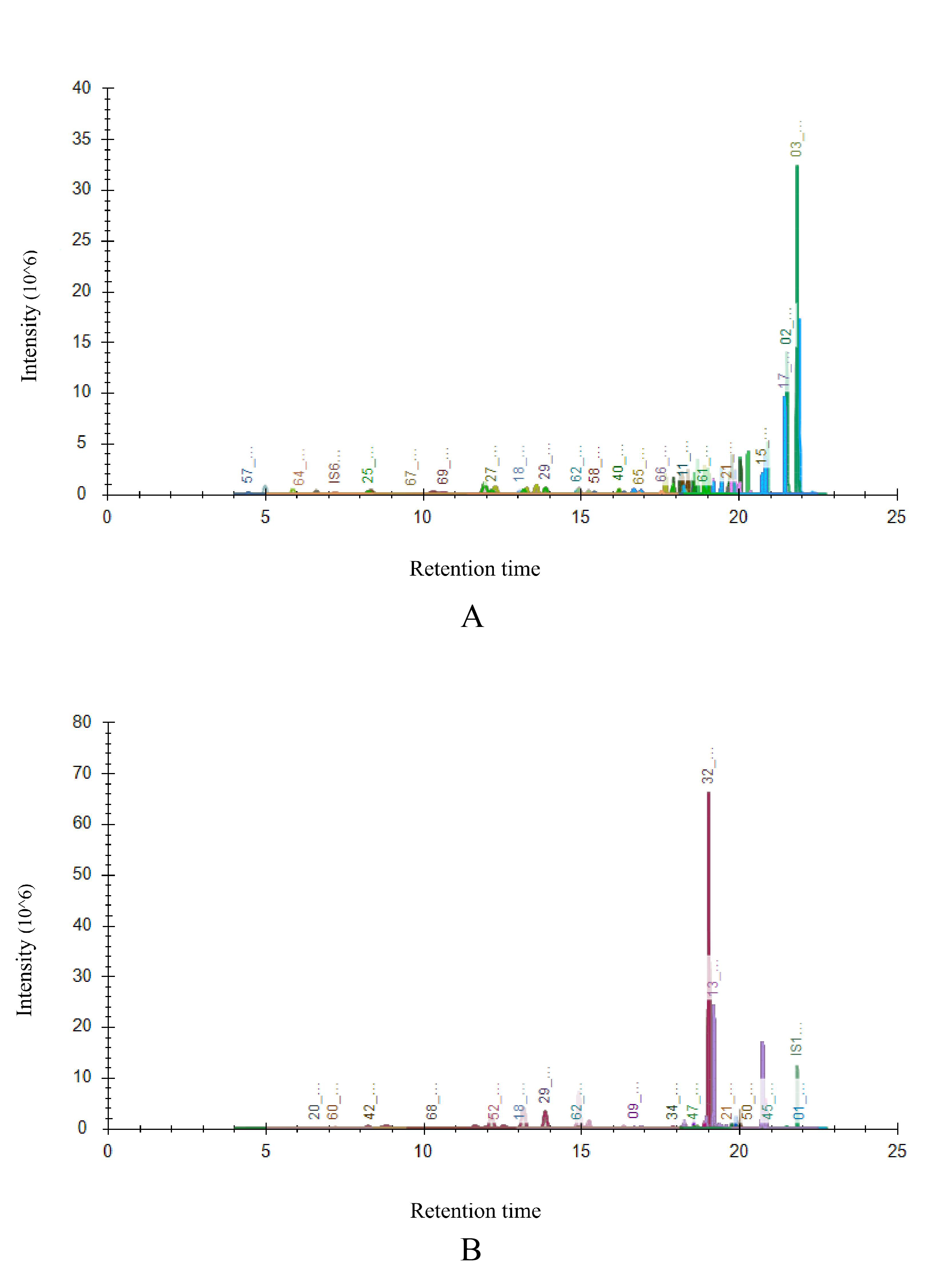

Supplement: Supplementary file 1 [file metabolites-14-00452-s001.zip › Figure S1.jpg]

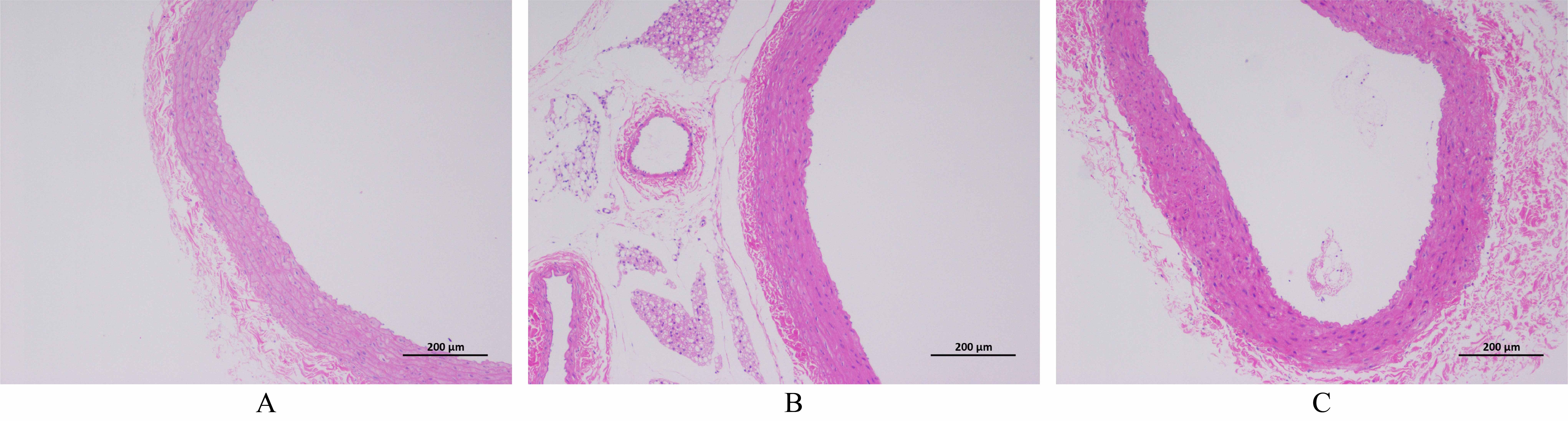

Supplement: Supplementary file 1 [file metabolites-14-00452-s001.zip › Figure S2.jpg]

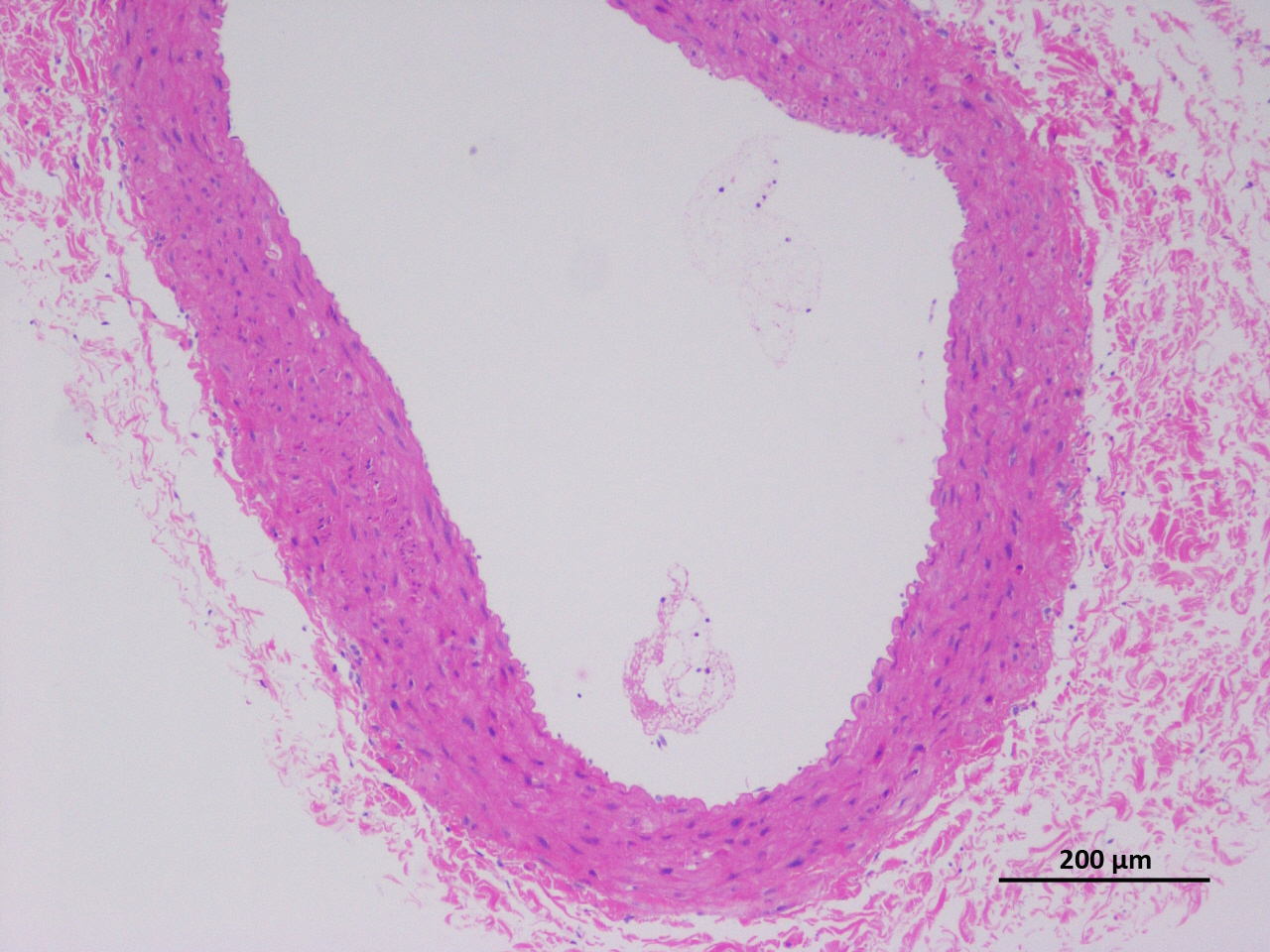

Supplement: Supplementary file 1 [file metabolites-14-00452-s001.zip › Raw Data/3.Artery histopathological observation/HE 10í┴10/HSD.jpg]

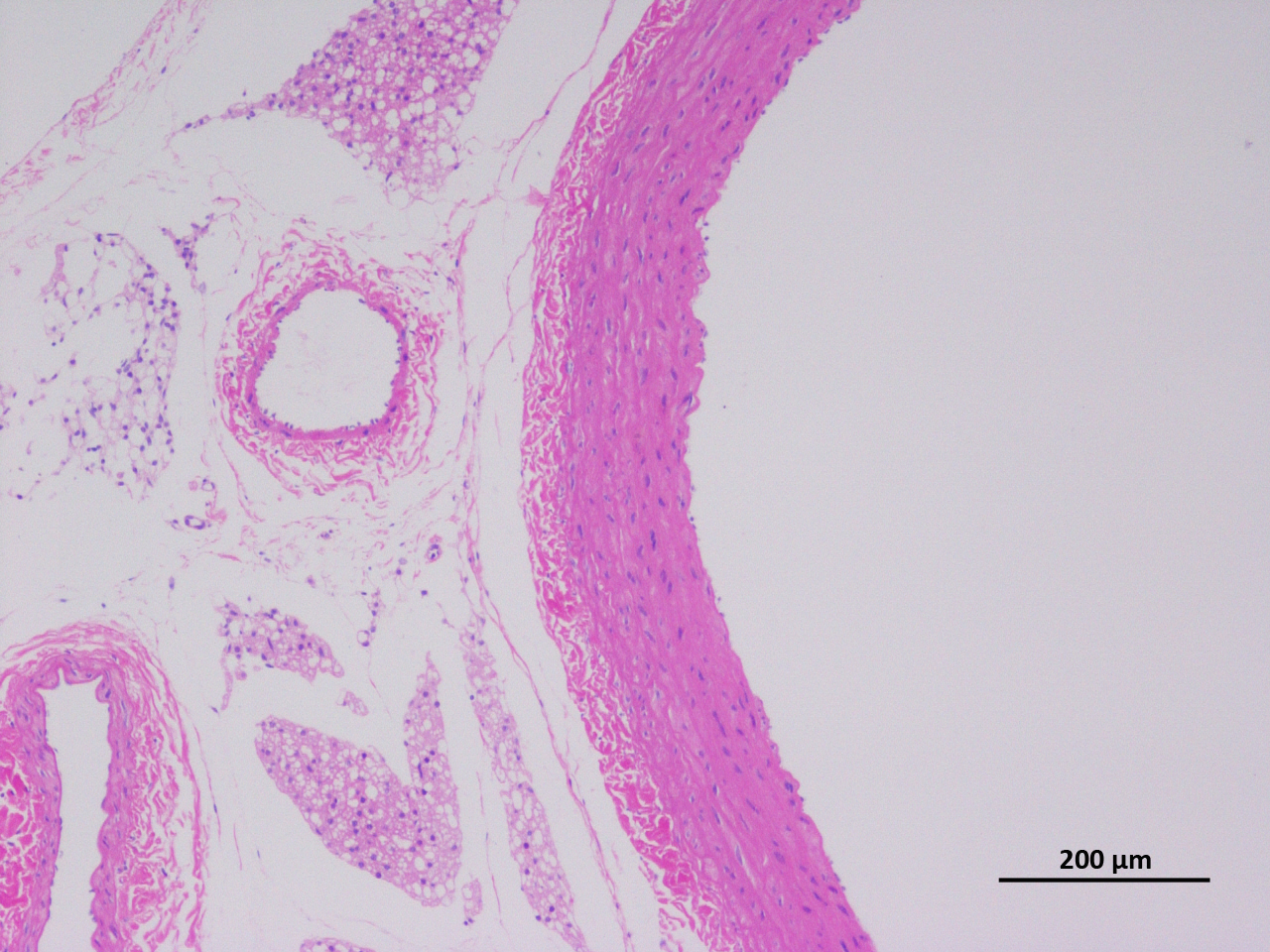

Supplement: Supplementary file 1 [file metabolites-14-00452-s001.zip › Raw Data/3.Artery histopathological observation/HE 10í┴10/LSD.jpg]

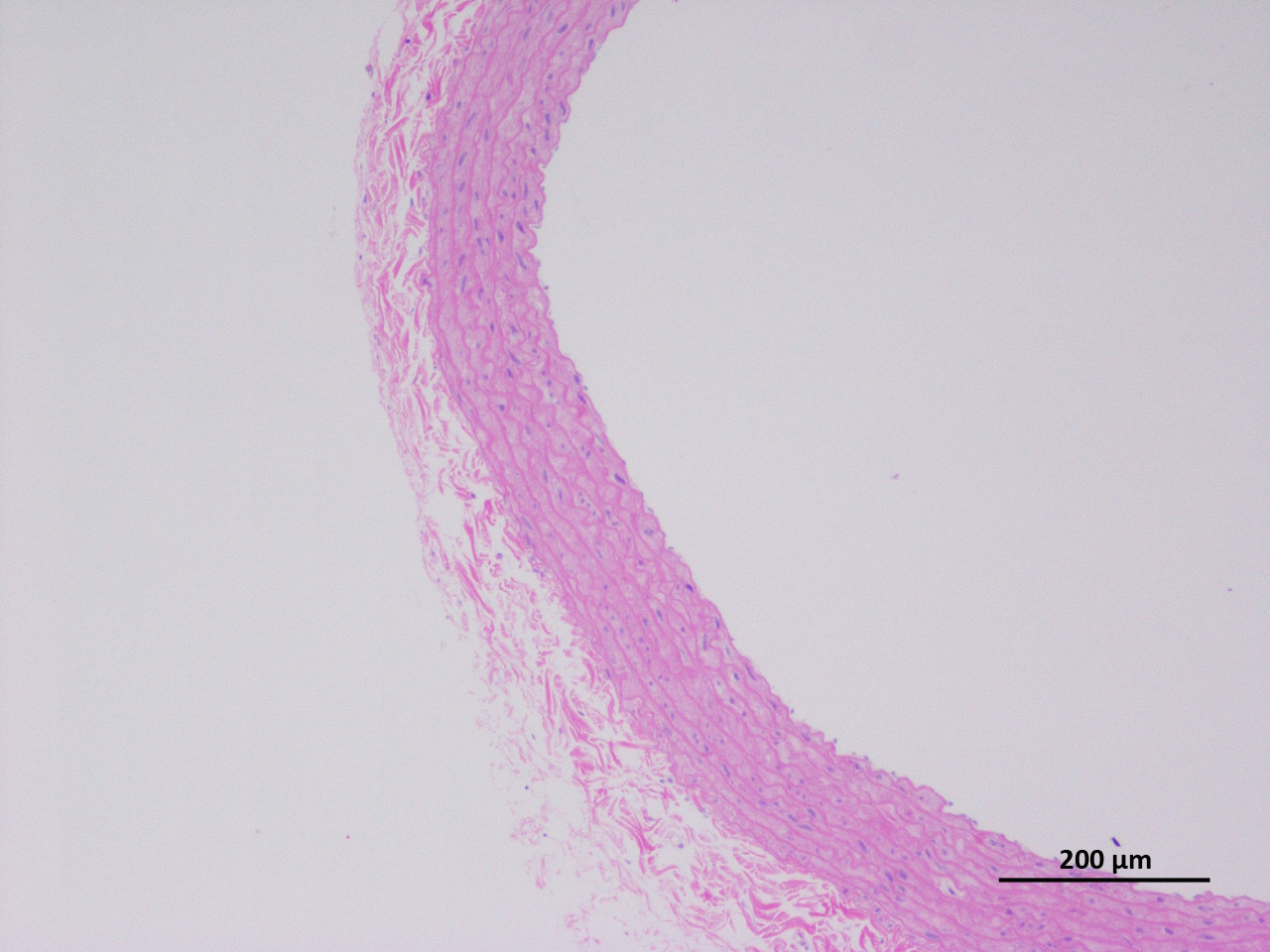

Supplement: Supplementary file 1 [file metabolites-14-00452-s001.zip › Raw Data/3.Artery histopathological observation/HE 10í┴10/NC.jpg]

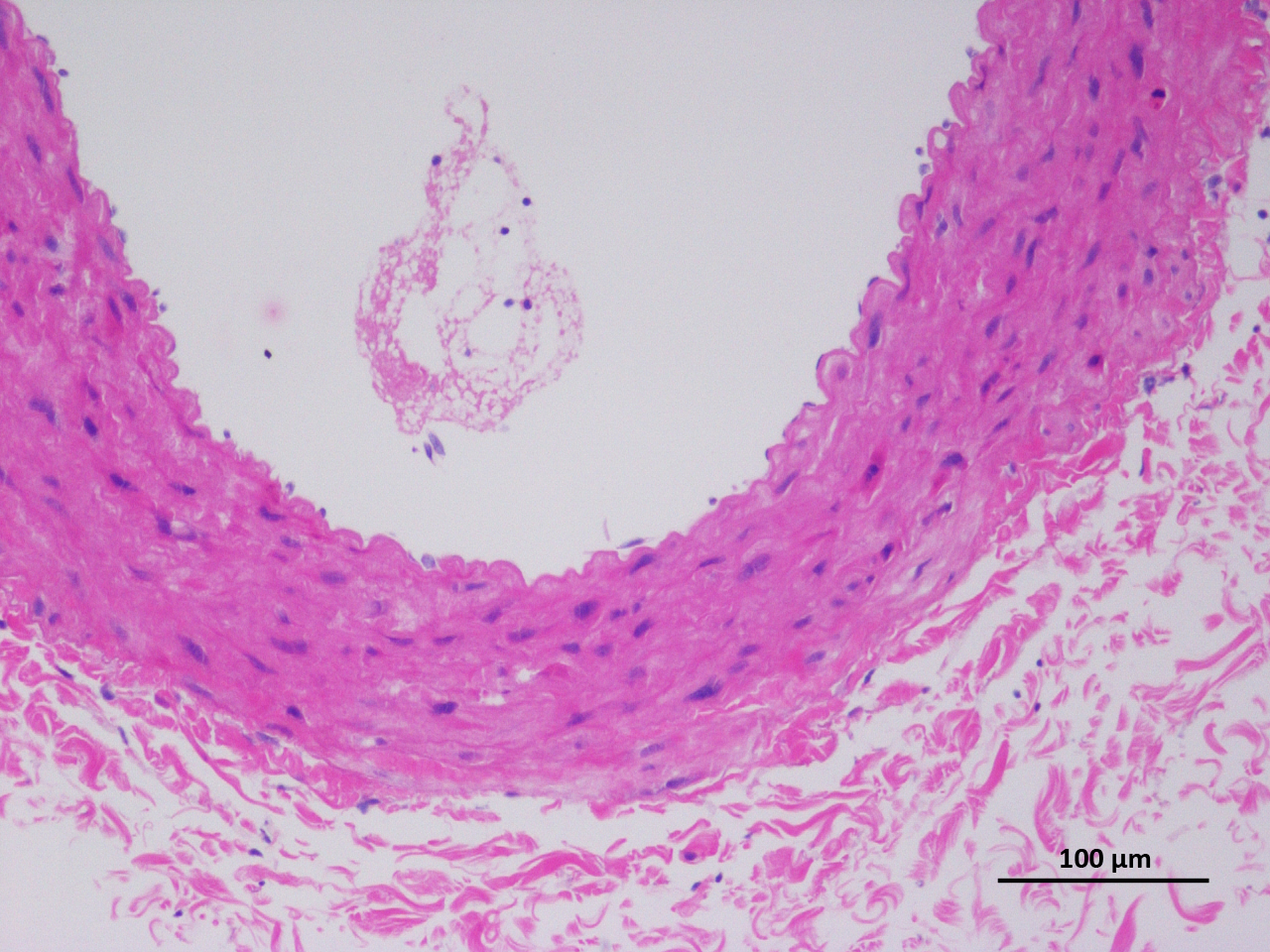

Supplement: Supplementary file 1 [file metabolites-14-00452-s001.zip › Raw Data/3.Artery histopathological observation/HE 20í┴10/HSD.jpg]

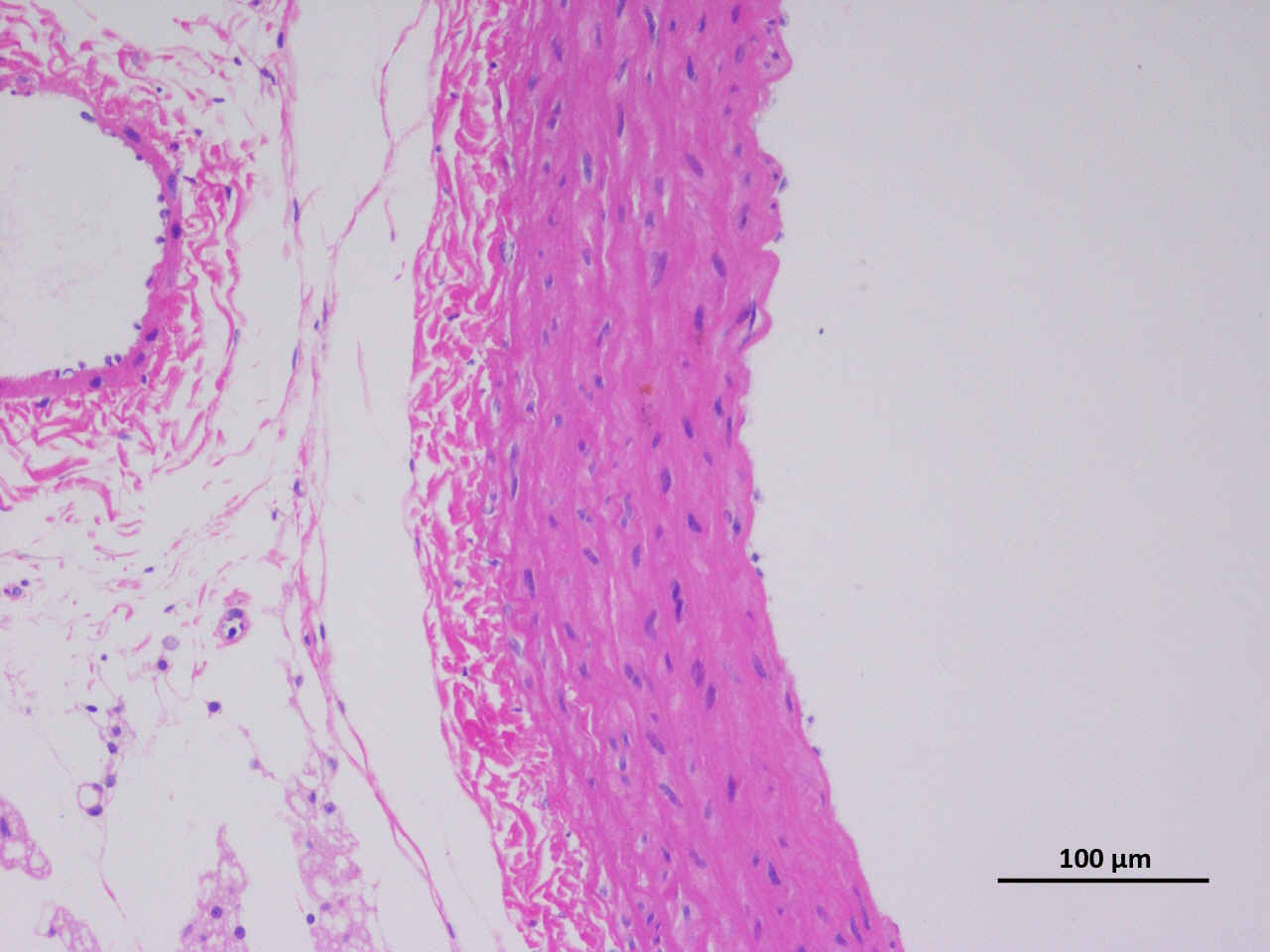

Supplement: Supplementary file 1 [file metabolites-14-00452-s001.zip › Raw Data/3.Artery histopathological observation/HE 20í┴10/LSD.jpg]

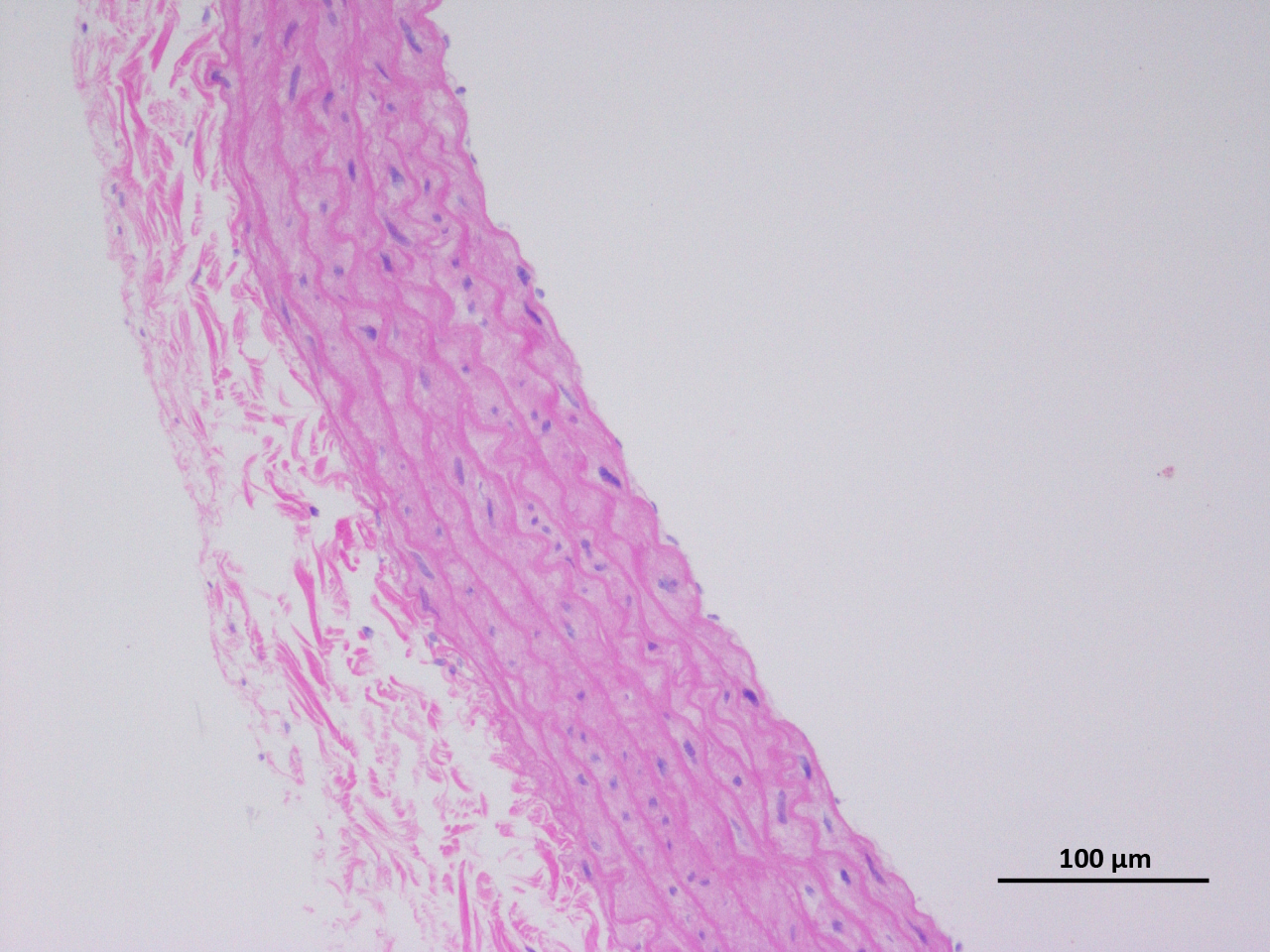

Supplement: Supplementary file 1 [file metabolites-14-00452-s001.zip › Raw Data/3.Artery histopathological observation/HE 20í┴10/NC.jpg]

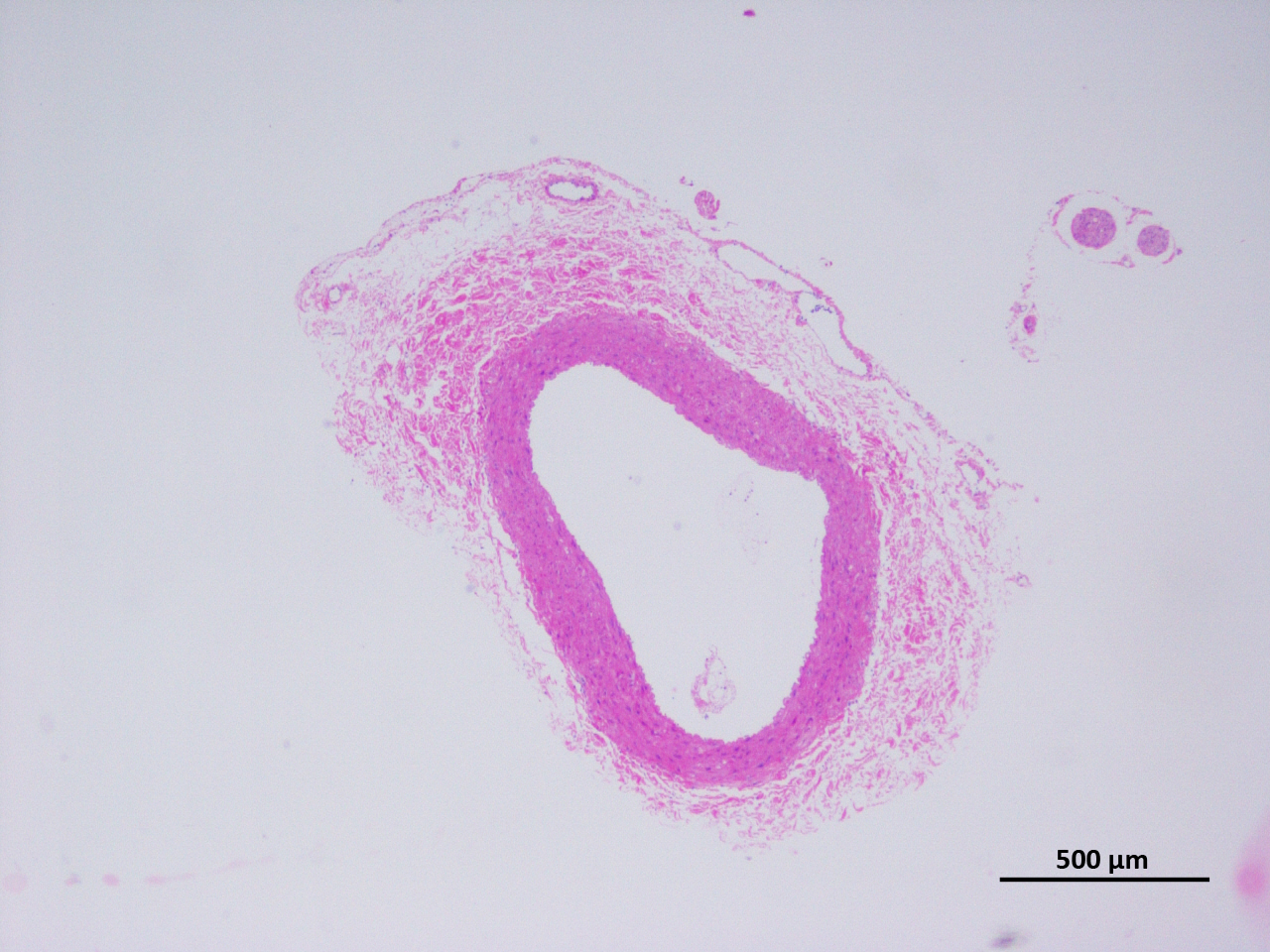

Supplement: Supplementary file 1 [file metabolites-14-00452-s001.zip › Raw Data/3.Artery histopathological observation/HE 4í┴10/HSD.jpg]

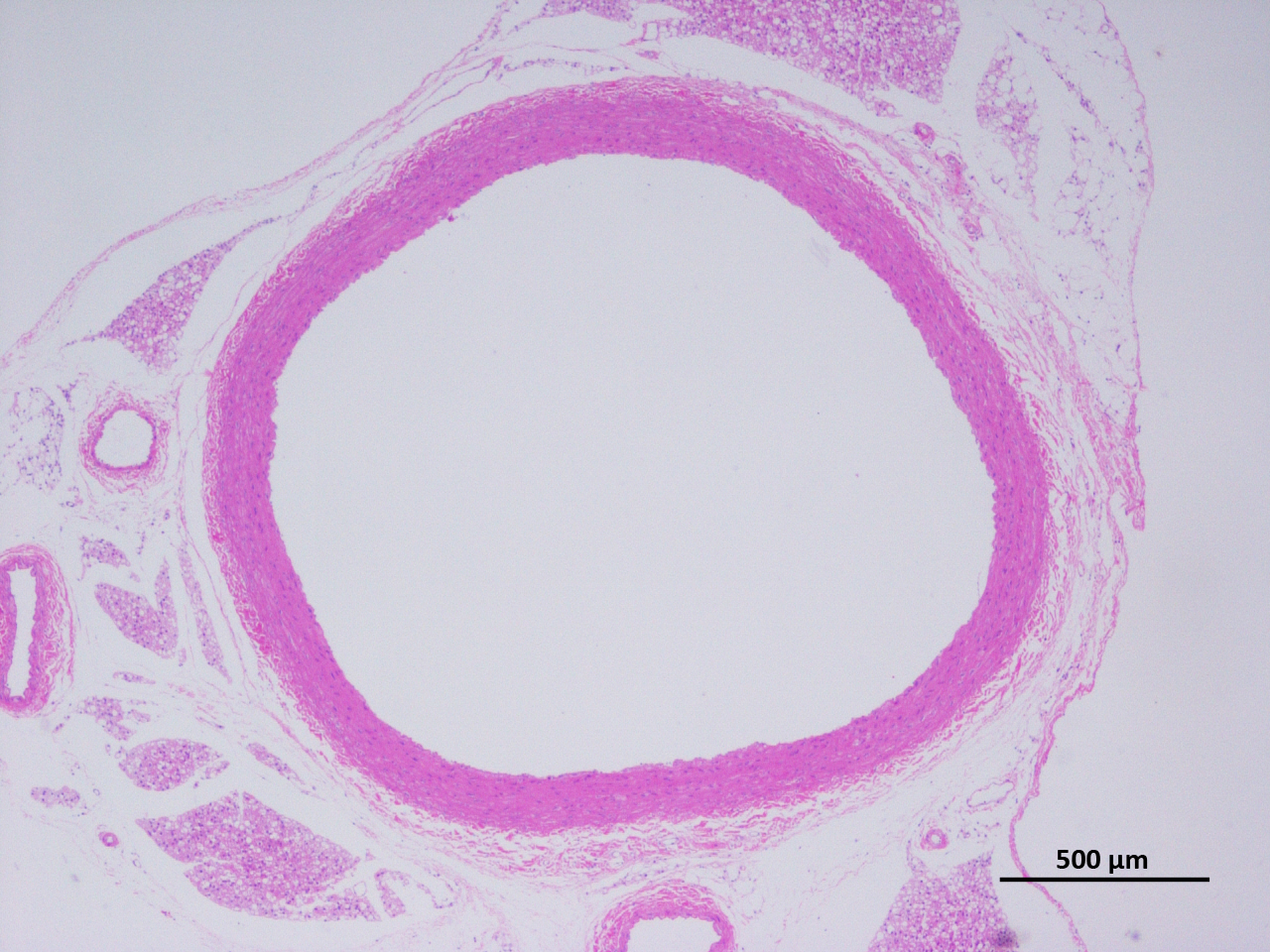

Supplement: Supplementary file 1 [file metabolites-14-00452-s001.zip › Raw Data/3.Artery histopathological observation/HE 4í┴10/LSD.jpg]

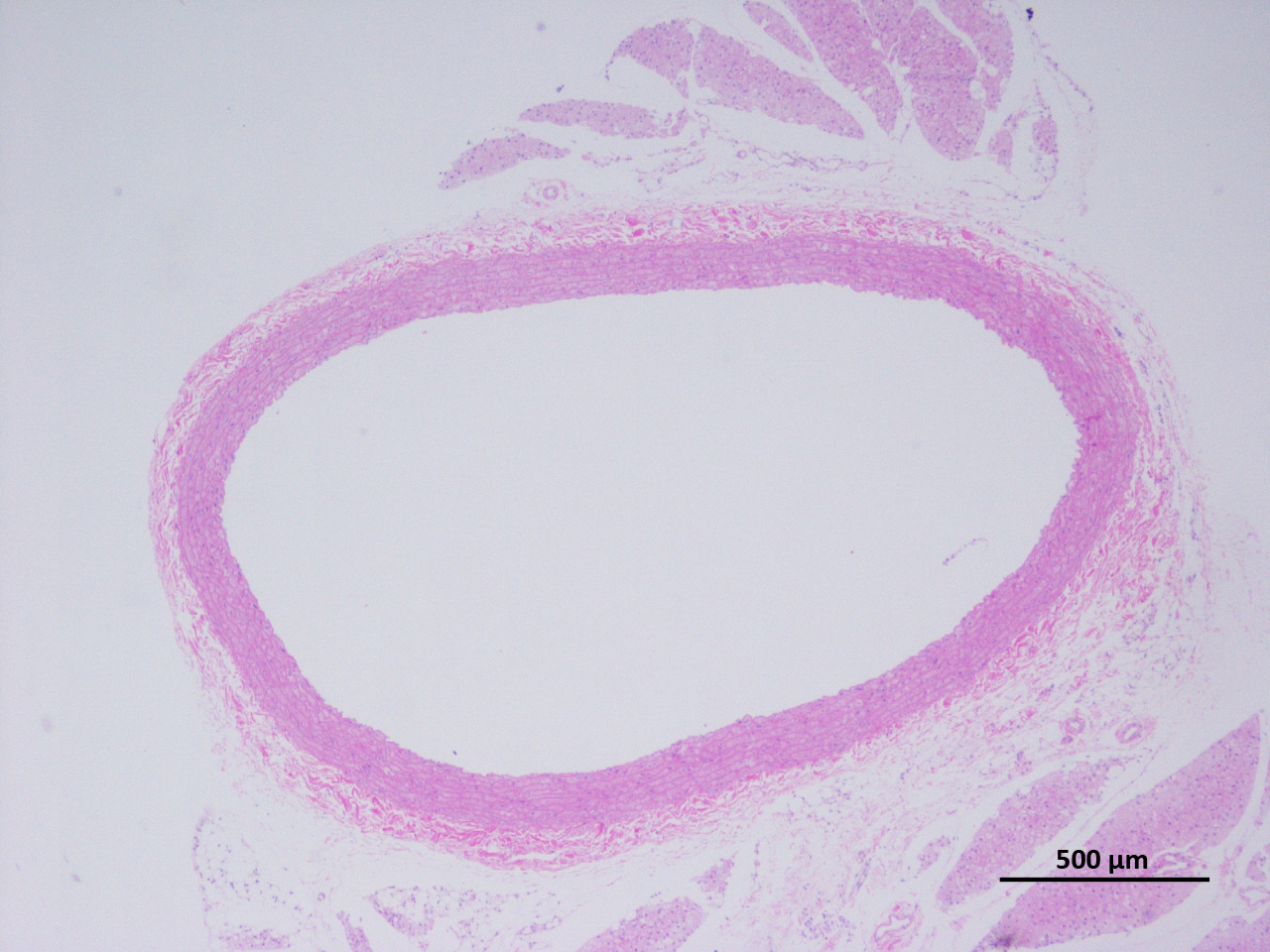

Supplement: Supplementary file 1 [file metabolites-14-00452-s001.zip › Raw Data/3.Artery histopathological observation/HE 4í┴10/NC.jpg]

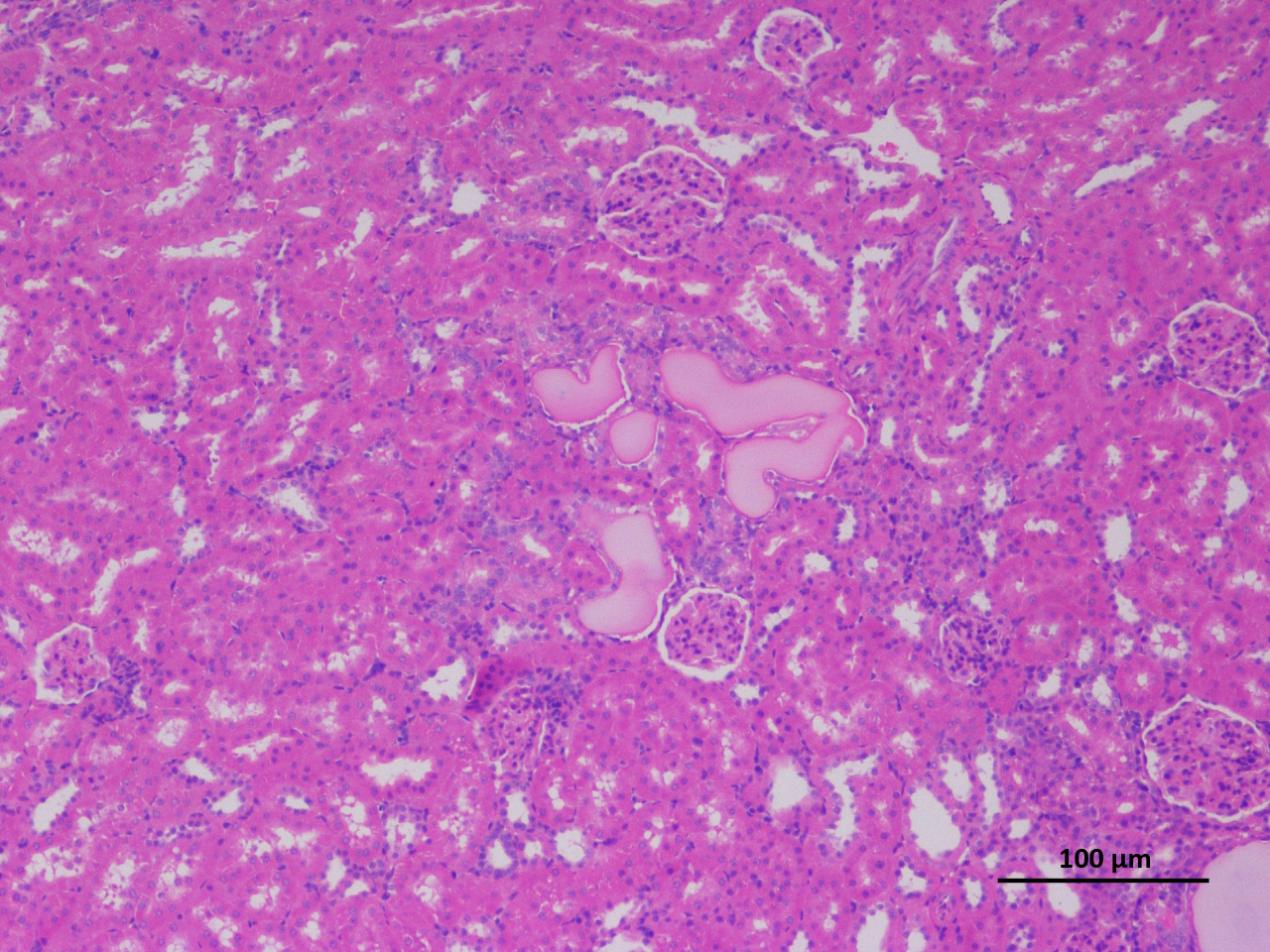

Supplement: Supplementary file 1 [file metabolites-14-00452-s001.zip › Raw Data/4.Renal histopathological observation/HE 10í┴10/HSD.jpg]

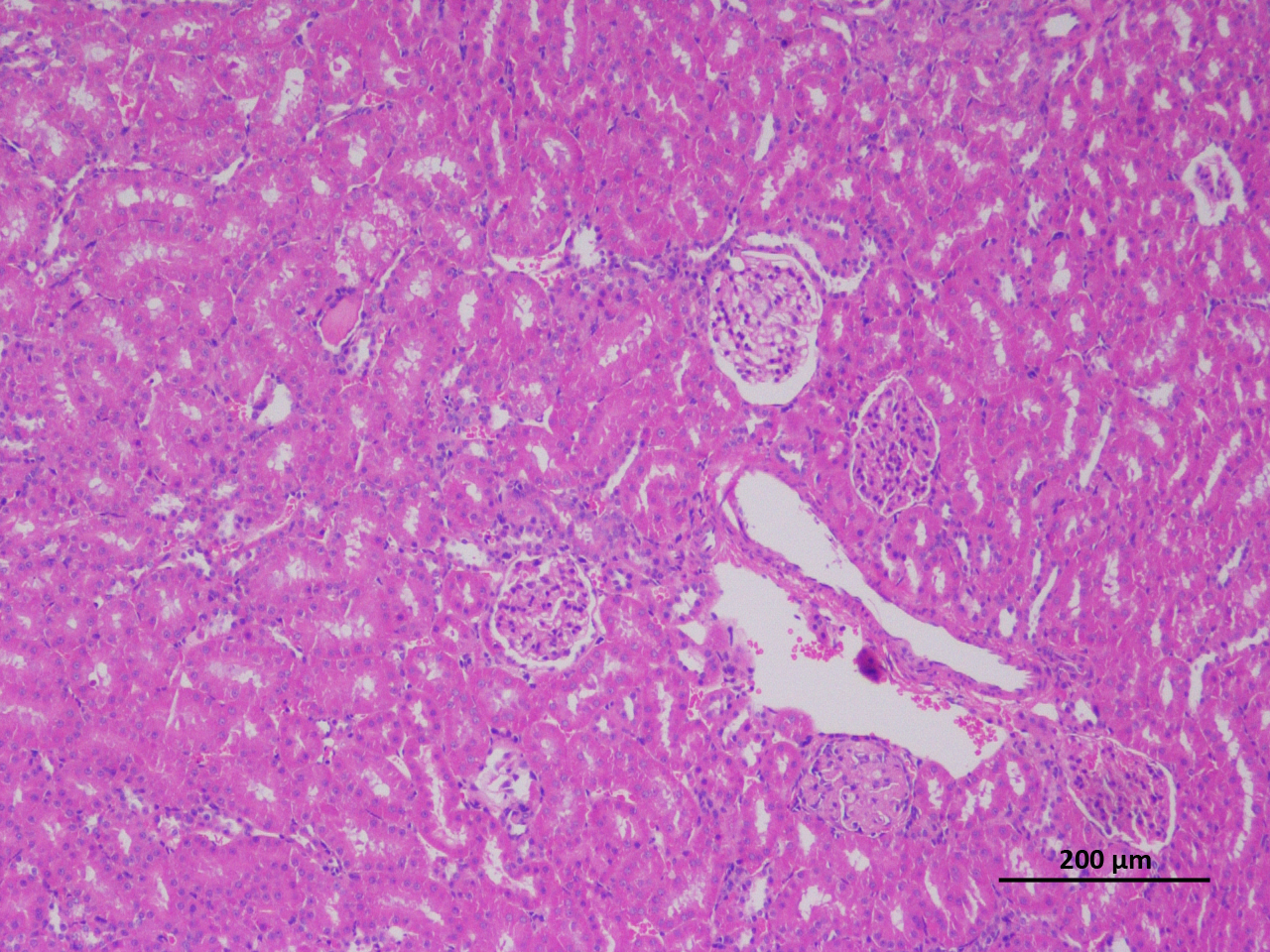

Supplement: Supplementary file 1 [file metabolites-14-00452-s001.zip › Raw Data/4.Renal histopathological observation/HE 10í┴10/LSD.jpg]

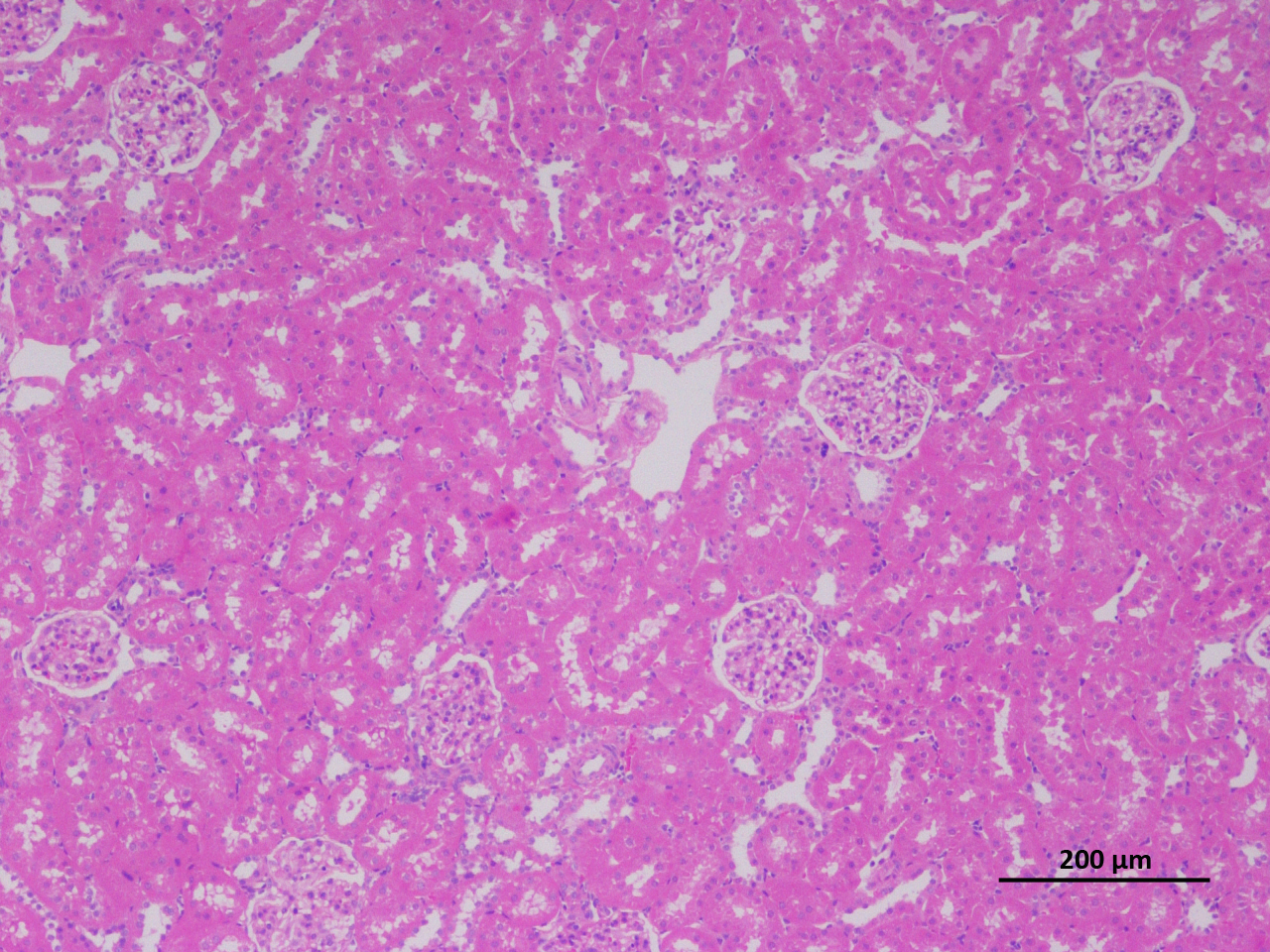

Supplement: Supplementary file 1 [file metabolites-14-00452-s001.zip › Raw Data/4.Renal histopathological observation/HE 10í┴10/NC.jpg]

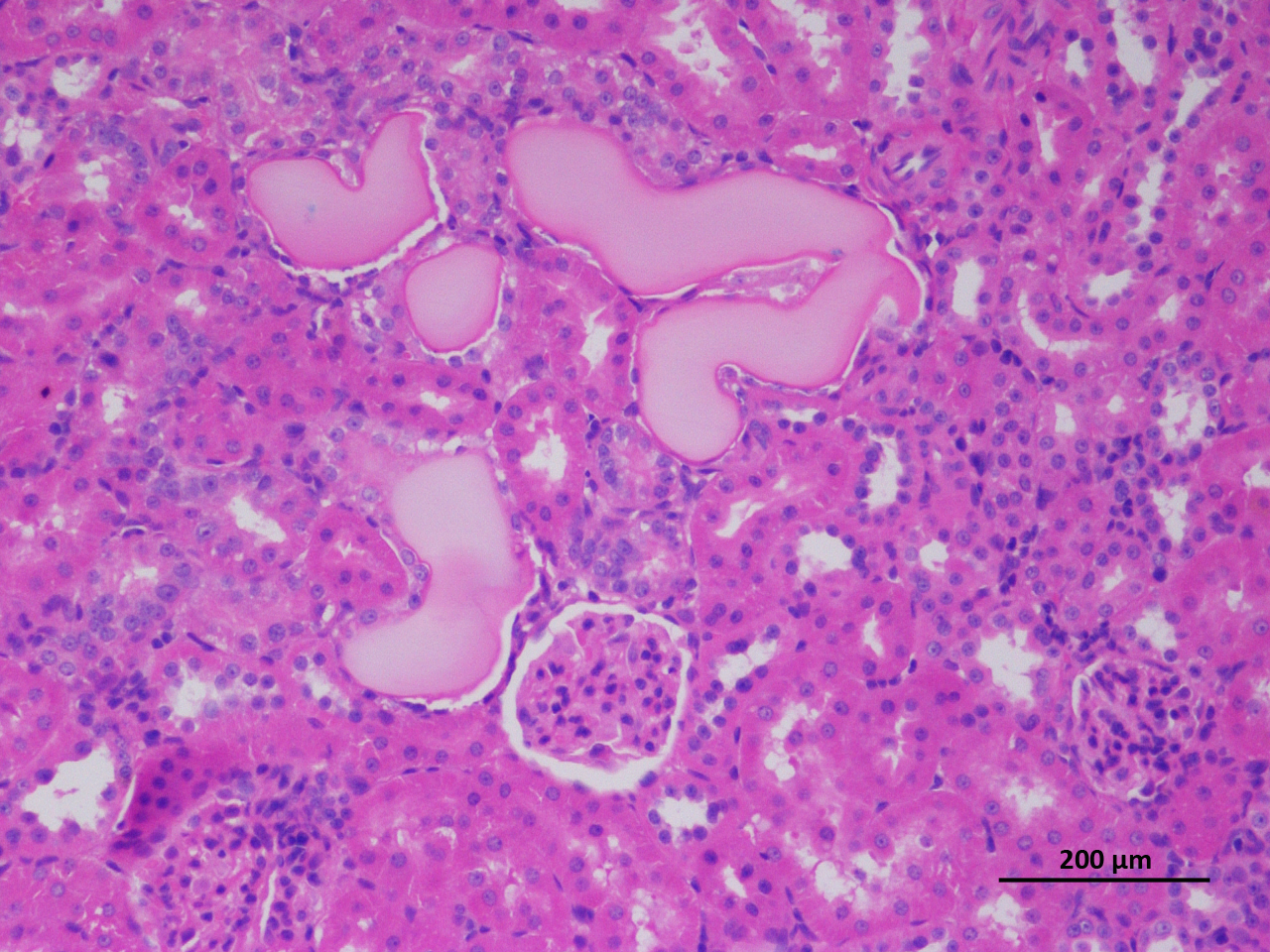

Supplement: Supplementary file 1 [file metabolites-14-00452-s001.zip › Raw Data/4.Renal histopathological observation/HE 20í┴10/HSD.jpg]

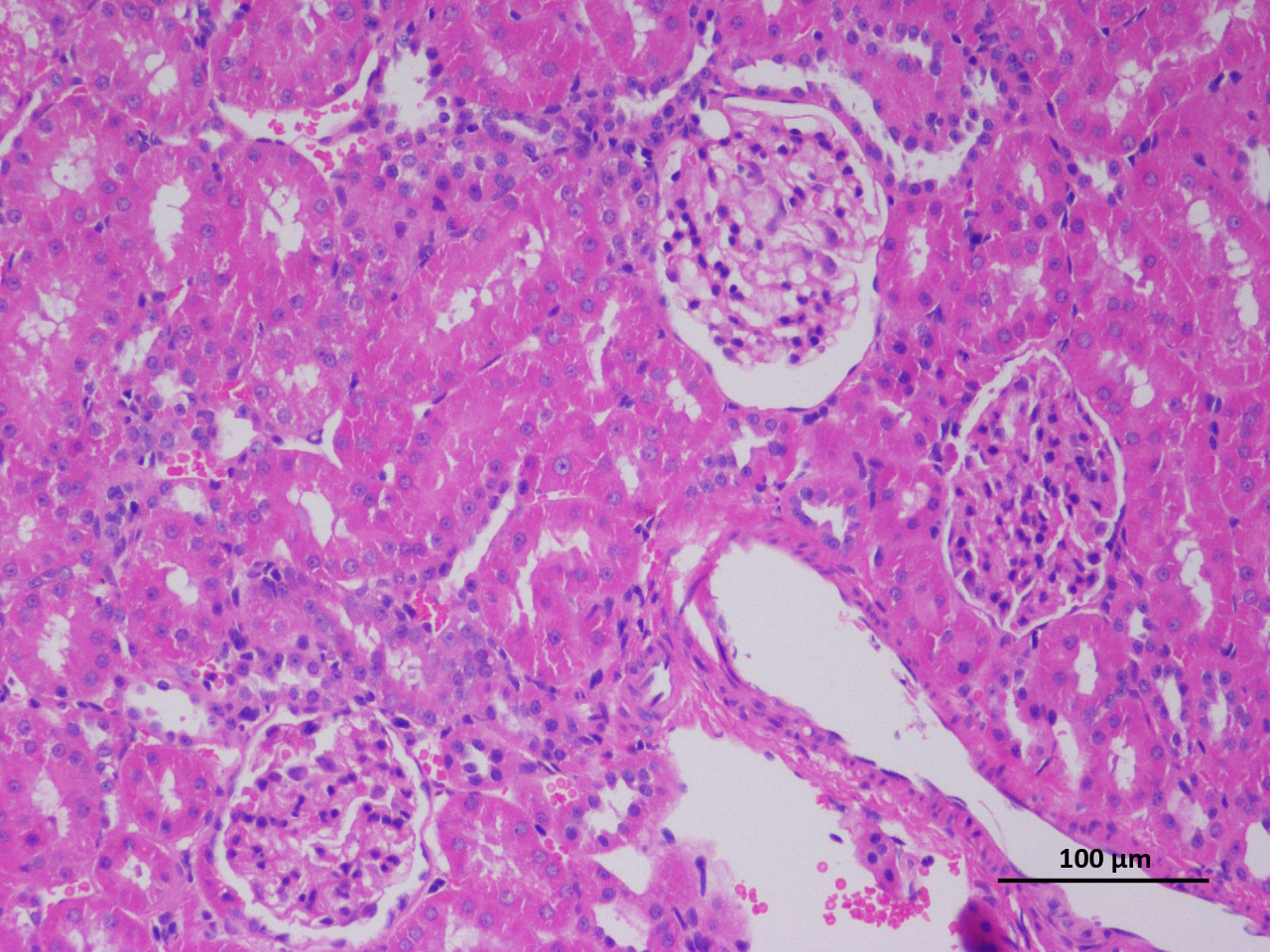

Supplement: Supplementary file 1 [file metabolites-14-00452-s001.zip › Raw Data/4.Renal histopathological observation/HE 20í┴10/LSD.jpg]

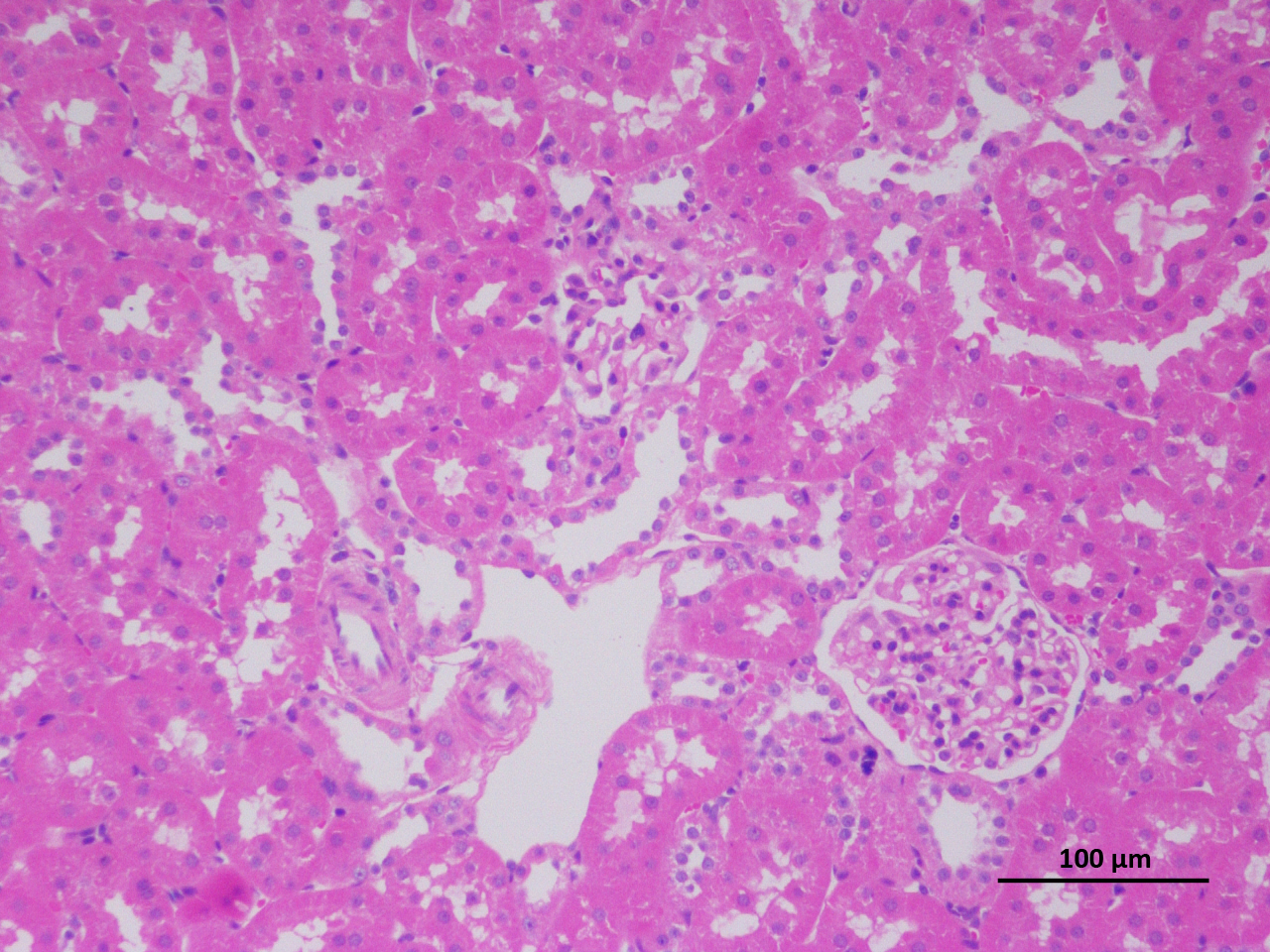

Supplement: Supplementary file 1 [file metabolites-14-00452-s001.zip › Raw Data/4.Renal histopathological observation/HE 20í┴10/NC.jpg]
